# Research Progress on Influencing Factors and Intervention Measures of Post-traumatic Growth in Breast Cancer Patients

**DOI:** 10.3389/fpubh.2022.927370

**Published:** 2022-06-21

**Authors:** Xin Fu, Jiao Sun, Xiaoxu Wang, Mingke Cui, Qiang Zhang

**Affiliations:** Department of Breast Oncology, Cancer Hospital of China Medical University, Liaoning Cancer Hospital and Institute, Shenyang, China

**Keywords:** breast cancer, post-traumatic growth, influencing factors, interventions, psychological health

## Abstract

Breast cancer is the highest incidence of female malignant tumor in the world, and it shows an increasing trend year by year. It poses a great threat to women's life and health and has become a public health issue of global concern. Paying attention to the psychological response of cancer patients is of definite value in helping patients cope with the disease, return to society, reshape an active and healthy life, and improve their quality of life with cancer. In recent years, researchers have increasingly focused on the positive changes experienced by cancer patients from the perspective of positive psychology, namely post-traumatic growth. It is of great significance to explore individual and social resources to help patients grow and improve their survival ability and quality of life by paying attention to the potential resources and positive forces in the process of patients' fighting against diseases. This paper summarizes the influencing factors and intervention measures of post-traumatic growth of breast cancer patients, providing ideas and reference for clinical medical staff to carry out relevant intervention.

Traumatic events refer to life threatening or serious injury events brought to human body by external factors beyond the scope of experience, causing physical or psychological damage to human beings, resulting in physical and mental troubles (1). Malignant tumors are a major global social burden that can significantly affect the mental health of patients and is considered a traumatic event that can cause psychological responses of fear, avoidance and hyperarousal (2). Among them, the diagnosis of breast cancer is a major traumatic event (3). Compared with classical trauma, its stressors are more complex, such as diagnosis of disease, severity and prognosis, treatment types, side effects, body image problems, loss of function and role changes in social life, etc. (4).

Breast cancer is a kind of physical and mental disease. In the past, people only pay attention to the patient's physical crisis, ignoring the psychological needs. With the development of positive psychology, some scholars have found positive psychological changes in cancer patients, such as the positive psychology of cherishing family love and health in fighting against disease, namely post-traumatic growth (PTG). The new concept of PTG was put forward by Tedeschi and Calhounn at the end of the twentieth century, which refers to the positive growth psychology of human beings after traumatic events. The proposal of this concept has shifted the research direction that has always been focused on post-traumatic negative psychology to post-traumatic positive psychology (5). Post-traumatic growth in cancer patients usually involves several aspects: new possibilities, close relationships, personal strength, spiritual development and greater appreciation for life (Figure 1) (6). PTG is a positive change resulting from fighting an extremely challenging life crisis (5).

**Figure 1:**
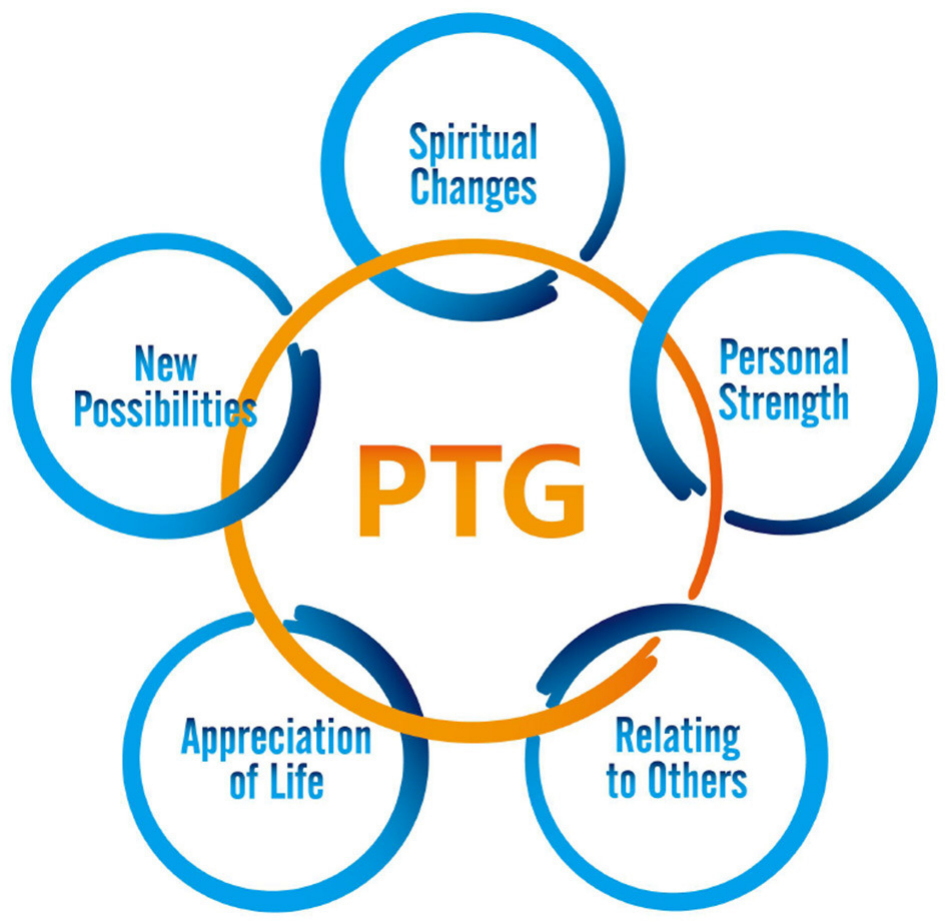
Forms of post-traumatic growth (PTG).

According to data from the American Cancer Society, there were 2.1 million breast cancer patients worldwide in 2018, accounting for 11.6% of the new cancer cases worldwide, and 6.6% of the new death cases, which has become a serious threat to human life and health. With the development of medical technology, the current 5-year survival rate after breast cancer treatment is about 80%, and the proportion of breast cancer survivors is increasing. Although the existing treatment methods can effectively alleviate the development rate of breast cancer, its psychological impact on patients cannot be ignored (7). Therefore, in order to better understand post-traumatic growth, this paper summarizes the influencing factors and intervention measures of post-traumatic growth in breast cancer patients, and actively discovers and excavates the positive growth of patients, which is of great significance to improve the health and quality of life of patients.

## Influencing Factors

The key predictors of PTG are the level of social support and the use of various coping strategies (8). Fujimoto and Okamura (9) conducted a study on 80 breast cancer patients aged 20–70 years, and conducted a single regression and multiple regression analysis on the situation of the patients. The results showed that positive coping, seeking emotional support and avoidance coping were the factors affecting PTG in breast cancer patients. Positive coping has the greatest impact because it is voluntary, linking cognition and action; Seeking emotional support is associated with close relationships, and women are more likely to use social support as a coping strategy when faced with stress; Avoidance coping involves not performing any particular action and engaging in an automatic, intrusive process of thinking and ruminating after the event. To alleviate suffering, PTG is said to arise through self-disclosure and self-analysis due to a variety of coping strategies, distraction, talking to people, and changing intrusive thoughts into more positive ones. Shi et al. (10) investigated 133 breast cancer patients in China. The results showed that binary coping and intimate relationship are the influencing factors of PTG, and strategies to improve the level of binary coping and avoid excessive intimate relationship between husband and wife are conducive to the occurrence of PTG in breast cancer patients. Romeo et al. (11) recruited 123 breast cancer patients to complete the investigation. The results showed that “fatalistic” coping strategies and perceived social support can improve patients' PTG level, and secure attachment style is a protective factor to prevent the development of anxiety and depression symptoms. Zhang (12) showed that self-transcendence and self-perceived burden were the influencing factors of PTG, and patients with high self-transcendence level and low self-perceived burden had higher PTG. Wang and Shi (13) found that clinical stage, surgical approach and postoperative complications were independent influences on PTG in 300 female breast cancer patients. Jia et al. (14) provided a survey of 303 breast cancer patients and found that self-care efficacy and psychological resilience were strongly and positively correlated with PTG. Mousavi and Vatankhah (15) found a significant correlation between religiosity, meaning of life and social support and PTG through a survey of 100 breast cancer patients, with social support being the best predictor.

## Interventions

### Narrative Nursing

Narrative nursing focuses on the construction of personal stories, constructed by telling life stories that connect past, present and future events and related cognition, emotion and self-expression to support patient PTG levels (16). Shi (17) adopted the narrative nursing process of “reception—assessment—narrative nursing—psychological intervention—follow-up” to conduct 4-week intervention for breast cancer patients. By exploring the patients' own strength, paying attention to humanistic care, and actively listening to and responding to the patients, the PTG level and cancer-related fatigue of the patients were improved, and the satisfaction of the patients was increases.

### Guided Nursing

Wang et al. (18) adopted nurse-led supportive intervention, designed according to patients' diagnosis, treatment process and needs, and focused on “becoming a patient,” “interpersonal relationship,” “recovery journey,” and “planning for the future” to guide patients to conduct research. The results suggested that the intervention has potential positive benefits of post-traumatic growth and relief of anxiety and depression in breast cancer patients.

### Family Dignity Intervention

Family dignity intervention is a psycho-psychological intervention strategy based on family caregivers. Through interviews with patients and caregivers, emotions are found that make patients feel value, dignity and meaning of life (19). Li et al. (20) set up a family dignity intervention team to implement family dignity intervention for breast cancer patients and their main caregivers, carrying out four stages of preparation, interview, editing and sharing, and providing targeted individual guidance. The results showed that this method can effectively reduce the burden of breast cancer patients care, and improve the level of post-traumatic growth.

### Acceptance and Commitment Therapy

Acceptance and commitment therapy, developed by Dr. Hayes and his research team at the University of Nevada, is a psychological and spiritual therapy for patients. It advocates acceptance of pain, including acceptance, cognitive dissociation, experience of the present, self-awareness, and action to clarify values and commitment (21). Sarizadeh and Mozaffari (22) used acceptance and commitment therapy to intervene in breast cancer patients, and the results showed that the fear of cancer recurrence and PTG in the experimental group were significantly improved. Ghorbani et al. (23) proved that acceptance and commitment therapy significantly improved mental flexibility, PTG and quality of life of breast cancer patients, and the improvement was significant over time.

### Mindfulness Therapy

Kriakous et al. (24) conducted Mindfulness-Based Stress Reduction (MBSR) intervention in 15 breast cancer patients, and the results showed that PTG levels and psychological capital were better than the control group, and the effect was maintained after 1-month follow-up. Mirmahmoodi et al. (25) conducted MBSR intervention on breast cancer patients for 8 weeks (once a week, 90 min each time), which significantly reduced patients' anxiety. Zhang et al. (26) used nurse-led mindfulness Taijiquan exercise plan to intervene breast cancer patients for 8 weeks (1 h each time, 2 times a week). The results showed that the method improved the level of PTG in patients, and the effect lasted for 1 year after intervention. The method was simple, effective and suitable for clinical nurses. Liu (27) established the mindful cancer rehabilitation group, which took mindfulness as the basis and combined the concepts of cancer knowledge and psychological knowledge to intervene for 8 weeks for breast cancer patients. The results showed that this method could significantly improve the level of PTG, reduce the anxiety and depression of patients, and relieve psychological stress. Carlson et al. (28) conducted a 12-month positive thinking-based cancer rehabilitation programme for 126 patients with stage I-III breast cancer. Results showed that this approach reduced fatigue, anxiety and confusion, relieved tension, sympathetic arousal and cognitive symptoms, improved PTG levels and had a sustained benefit over 1 year. Norouzi et al. (29) conducted a positive thinking based cognitive therapy intervention in 20 breast cancer patients over a total of 8 sessions over 2 months. Results showed that this approach increased PTG levels and improved aspects of self-management and functional impairment.

### PTG Plan

Choi et al. (30) used the PTG plan to intervene in breast cancer patients, including writing and self-reflection for self-analysis, conversation and sharing for self-disclosure, and participation in self-help groups for social support. Compared with the control group, the experimental group had higher PTG scores and lower emotional distress, suggesting that the PTG plan improved THE PTG of women with breast cancer.

### Cognitive Emotion Intervention

Hamidian et al. (31) used emotional cognitive training to intervene breast cancer patients for 5 times, twice a week, and collected PTG of patients 20 weeks after the last cognitive intervention. It is found that cognitive emotion intervention has a positive and significant effect on post-traumatic growth in women with breast cancer, and can be used as a way to promote PTG in women undergoing breast cancer treatment.

### Psycho Educational Group

Masoum et al. (32) used the psycho educational group intervention method to treat 80 Iranian breast cancer patients, and taught the course content of health education, stress management, relaxation training and coping skills twice a week, 90 min each time, six times in total. The results showed that this method could improve the level of PTG, and psychological intervention such as psychological education had positive effects on traumatic growth, subjective wellbeing and treatment compliance. Hashemi and Raghibi (33) conducted an 8-week structured group therapy with 11 patients with breast cancer treated with chemotherapy and radiotherapy. Patients received a series of positive psychology exercises focusing on how to increase new possibilities, personal strength, appreciation of life, using empowerment exercises to build relationships with others, expressing positive things, gratitude, enjoying each day's experience, responding positively and taking stock of life, and the results found that the approach accelerated PTG and was of greater interest to patients with higher education.

### Expressive Writing

Expressive writing is considered to be an effective way to manage stress or traumatic events, leading to beneficial cognitive change and more adaptive behavior (34). In particular, expressive writing is thought to use writing processes to construct coherent narratives (35), which involves written disclosure of thoughts and feelings about traumatic events, thereby enhancing cognitive processing and improving stress regulation (36). Cognitive words refer to words related to cognitive processing, including insightful words (e.g., “aware”) and causal words (e.g., “because”) (35). Meaning construction refers to the process of trying to understand stressful events within the current framework of meaning and reconstructing one's beliefs to understand these events. In the meaning construction model, meaning in life is regarded as the result of meaning construction (37). The use of cognitive words in expressive writing facilitates the process of meaning construction, enabling individuals to understand stressful events and achieve PTG. Zheng et al. (38) gave 52 adolescents a 20-min expressive writing task to write down traumatic events in detail. It was found that expressive writing and the use of causal and insightful words contributed to the generation of meaning in the writing process and increased levels of PTG. Therefore, the use of causal and insightful words in expressive writing increases the effectiveness of interventions by promoting meaning construction and PTG, and evaluating the content of expressive writing helps clinicians determine the stages of meaning creation and the individual's level of success in actual meaning creation. Gallagher et al. (39) selected 96 Chinese American breast cancer patients and used expressive writing intervention to ask patients to write about cancer-related topics, which were divided into three groups: writing about cancer experience facts, emotional disclosure or self-regulation, and found that PTG could be promoted.

## Discussion

At present, domestic research on PTG is in its infancy, mostly focusing on cancer. Among them, the psychological stress of breast cancer patients mainly comes from fear of disease recurrence, body dysmorphic disorder, chemotherapy, surgery, young age, high degree of disease, lack of social support and other factors. Stress, depression, anxiety and other psychological reactions are related to the diagnosis and treatment of diseases. Psychological problems will not only damage the patient's immune system, but also seriously affect their quality of life. Coping style, social support and personal characteristics are the key factors influencing post-traumatic growth. Interventions in recent years include narrative nursing, guided nursing, family dignity intervention, acceptance and commitment therapy, mindfulness therapy and PTG plan. In-hospital nursing and out-hospital extended nursing developed together. It mainly focuses on personal emotional catharsis, family support, social support and other aspects of breast cancer patients, so that patients can find their dignity and get hope and enthusiasm for life, so as to improve the level of PTG, face the disease with positive psychology, and improve the quality of life. In clinical application, innovative and highly targeted, operable and extendible nursing measures should be constructed according to the specific conditions of patients. Combined application can also be considered, and further research should be carried out on PTG (Table 1).

**Table 1:** Post-trauma interventions for breast cancer.

**Interventions**	**Mechanism of action**
Narrative nursing	Improving PTG
Guided nursing	
Family dignity intervention	
Acceptance and commitment therapy	
Mindfulness therapy	
PTG plan	
Cognitive emotion intervention	
Psycho educational group	
Expressive writing	

## Author Contributions

XF wrote the manuscript. XW and MC contributed to design, acquisition, analysis, and interpretation of data. JS reviewed the manuscript. QZ contributed to the conception of review and approved the final version. All authors contributed to the article and approved the submitted version.

## Conflict of Interest

The authors declare that the research was conducted in the absence of any commercial or financial relationships that could be construed as a potential conflict of interest.

## Publisher's Note

All claims expressed in this article are solely those of the authors and do not necessarily represent those of their affiliated organizations, or those of the publisher, the editors and the reviewers. Any product that may be evaluated in this article, or claim that may be made by its manufacturer, is not guaranteed or endorsed by the publisher.
